# Transjugular intrahepatic portosystemic shunt creation (TIPS) in the angio-CT—a hybrid intervention with image fusion

**DOI:** 10.1007/s00330-023-09793-9

**Published:** 2023-06-07

**Authors:** Jonathan Nadjiri, Tobias Waggershauser, Marc Mühlmann, Ursula Ehmer, Fabian Geisler, Ulrich Mayr, Tobias Geith, Philipp M. Paprottka

**Affiliations:** 1 Department of Interventional Radiology, School of Medicine, University Hospital Klinikum Rechts Der Isar TUM, Technical University of Munich, Ismaninger Straße 22, 81675 Munich, Germany; 2 Department of Medicine II, School of Medicine, Technical University of Munich, University Hospital Klinikum Rechts Der Isar, Munich, Germany

**Keywords:** Portasystemic shunt, Transjugular intrahepatic, Angiography, CT, Hypertension, Portal

## Abstract

**Objective:**

For transjugular intrahepatic portosystemic shunt (TIPS) creation, ultrasound guidance for portal vein puncture is strongly recommended. However, outside regular hours of service, a skilled sonographer might be lacking. Hybrid intervention suites combine CT imaging with conventional angiography allowing to project 3D information into the conventional 2D imaging and further CT-fluoroscopic puncture of the portal vein. The purpose of this study was to assess whether TIPS using angio-CT facilitates the procedure for a single interventional radiologist.

**Methods:**

All TIPS procedures from 2021 and 2022 which took place outside regular working hours were included (*n* = 20). Ten TIPS procedures were performed with just fluoroscopy guidance and ten procedures using angio-CT. For the angio-CT TIPS, a contrast-enhanced CT was performed on the angiography table. From the CT, a 3D volume was created using virtual rendering technique (VRT). The VRT was blended with the conventional angiography image onto the live monitor and used as guidance for the TIPS needle. Fluoroscopy time, area dose product, and interventional time were assessed.

**Results:**

Hybrid intervention with angio-CT did lead to a significantly shorter fluoroscopy time and interventional time (*p* = 0.034 for both). Mean radiation exposure was significantly reduced, too (*p* = 0.04). Furthermore, the mortality rate was lower in patients who underwent the hybrid TIPS (0% vs 33%).

**Conclusion:**

TIPS procedure in angio-CT performed by only one interventional radiologist is quicker and reduces radiation exposure for the interventionalist compared to mere fluoroscopy guidance. The results further indicate increased safety using angio-CT.

**Clinical relevance statement:**

This study aimed to evaluate the feasibility of using angio-CT in TIPS procedures during non-standard working hours. Results indicated that the use of angio-CT significantly reduced fluoroscopy time, interventional time, and radiation exposure, while also leading to improved patient outcomes.

**Key Points:**

• *Image guiding such as ultrasound is recommended for transjugular intrahepatic portosystemic shunt creation but might be not available for emergency cases outside of regular working hours.*

• *Transjugular intrahepatic portosystemic shunt creation using an angio-CT with image fusion is feasible for only one physician under emergency settings and results in lower radiation exposure and faster procedures.*

• *Transjugular intrahepatic portosystemic shunt creation using an angio-CT with image fusion seems to be safer than using mere fluoroscopy guidance.*

## Objective

Transjugular intrahepatic portosystemic shunt (TIPS) procedure outside regular working hours is associated with a high mortality [1, 2]. In general, it is recommended that TIPS is performed under sonographic guidance [3, 4]. However, especially in emergency situations outside of regular duty hours, there may not be an additional experienced physician available to provide sonographic guidance [5]. Therefore, in practice, the interventional radiologist may have to perform the procedure under mere angiographic guidance and alone. This circumstance may lead to higher mortality. Angio-CTs have been entering the market for some time [6]. These devices combine a CT with an angiography unit. Interventions can thus be performed multimodally on one patient. No repositioning is required when changing modalities. Switching between the two modalities can be done quickly [7–10]. This way, the images and information generated in the CT can be superimposed directly on the angiographic display monitor. These landmarks can then be used for interventions. It is also possible, for example, to produce a portal venous CT as basis for the planned procedure. From this, a 3D model of the portal vein can be created using virtual rendering technique (VRT). Then, a landing zone for the TIPS needle can be marked by directly transferring the 3D data in the “live” angiography images [11, 12]. In this work, we would like to investigate the extent to which the TIPS procedure performed in hybrid mode with an angio-CT facilitates a single interventional radiologist under emergency conditions. We hypothesized that the TIPS procedure in hybrid mode using angio-CT results in a shortened overall procedure time because navigation points are already set. Therefore, we also believe that it will decrease radiation exposure for the interventional team.

## Methods

### Ethical approval

This retrospective study design was approved by the local ethics committee. Study consent was waived by the local ethics committee. All procedures performed in studies involving human participants were in accordance with the ethical standards of the institutional and national research committee and with the 1964 Helsinki declaration and its later amendments or comparable ethical standards.

### Study population

Between January 2021 and December 2022, all patients who received a TIPS procedure that took place outside of regular service hours were retrospectively enrolled in this study. It was recorded whether the TIPS procedure was performed in a hybrid technique or in a conventional solely fluoroscopically guided manner.

### Procedure and angio-CT

#### Angio-CT

The angio-CT used is a Siemens nexaris system (nexaris Angio-CT, Siemens Healthcare GmbH). The CT is one hundred and twenty-eight, slice device. The entire system is a two-room-solution allowing for both devices being operated separately. The gantry is oriented towards the angiography table.

#### Protocol

The patient is positioned in the supine position. The head is positioned in the direction of the CT side to allow easy transjugular access from behind the head. Positioning is as close as possible to the upper end or CT side of the table. A portal venous contrasted CT was initially performed for the hybrid TIPS planning. Therefore, 70–90 mL of contrast agent was administered (Imeron 400; Bracco) at 2 mL/s followed by a 50-ml saline chaser at the same injection speed using an automated dual-syringe power injector (Medtron, ACCUTRON® CT). The scan parameters were as follows: single-energy mode with activated automatic attenuation-based tube current modulation (CareDose4D), automatic kV modulation (CareKV), gantry rotation: 0.28 s; pitch: 1.9, collimation 0.6 × 192 mm, scan delay was 70 s. While the interventional radiologist performs post-processing, the patient is sterilely draped and prepared for the procedure. Post-processing and creation of the VRT is done at the Syngo Leonardo workstation (syngo 2005A (VD30A), Siemens Healthcare GmbH). Bones are segmented at the workstation, as well as the following markers were set: most favorable hepatic vein, optimal portal vein entry point, and inferior hepatic boarder. This information or VRT, respectively, is then transferred to the live image angiography monitor using image fusion.

#### Interventional procedure

**Fig. 1:**
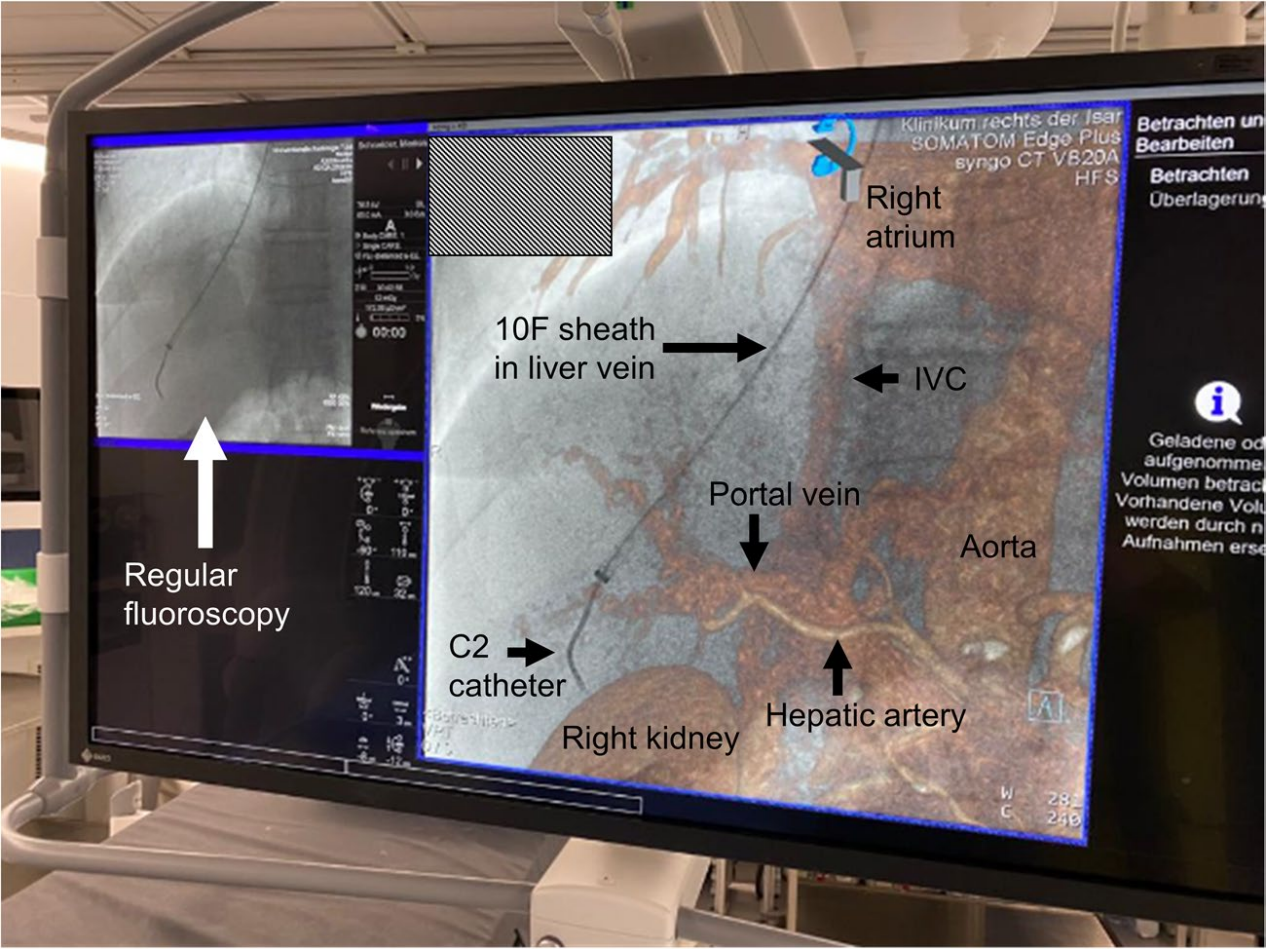
Here the view and perspective of the interventional radiologist during hybrid TIPS is shown. The image sports a fusion of virtual rendering technique and conventional fluoroscopy. A 10F sheath is inserted via a C2 catheter into the right liver vein to prepare portal vein puncture. (IVC, inferior vena cava)

All TIPS procedures included in this study were performed by the same interventional radiologist. The procedures were conducted under sterile conditions. The procedures are usually performed under analgesia or general anesthesia. If there is no general anesthesia and there is no right jugular central venous catheter, first local anesthesia is performed using 10 ml Scandicain cutaneous and subcutaneously, then ultrasound-guided (Freestyle, Siemens Healthcare GmbH) puncture of the right internal jugular vein using Abbocath 18 G, advancement of a 35-gauge hydrophilic wire (Radifocus™ Guide Wire M Standard Type, Terumo), insertion of a 10F sheath (Flexor, Cood medical), probing of the hepatic vein using C2 4F catheter (Tempo™, Cordis™), and advancement of the sheath via a stiff wire if necessary (Amplatz Super Stiff™, Bosten Scientifc). If the procedure was performed in hybrid mode (angio-CT), the placement of the sheath as well as the TIPS needle is performed in the previously selected most favorable vein. From the vein, the needle is then inserted in the direction of the portal vein as displayed on the live angiography monitor during the hybrid procedure. Marking the inferior border of the liver is particularly helpful to avoid punctures of the liver capsule from within the liver. Figure 1 illustrates the view from the perspective of the interventional radiologist. If the interventions guided by mere fluoroscopy orientation is based on anatomical landmarks and in conjunction with an ultrasound examination performed directly before the start of the intervention and, if available pre-existing CT and MRI images, after successful puncture of the portal vein, the guidewire is advanced and the C2 catheter is guided into the portal venous system. From this point, in the angio-CT group, image fusion is switched off or superimposition of 3D CT data is switched off, respectively. Then the Amplatz wire and a 5-F measuring pigtail catheter were inserted (Accu-Vu, aimecs® GmbH). Pressure measurement is also performed via the pigtail catheter using a riser tube in the portal venous system and the central venous system. Length determination for the stent (VIATORR®, GORE®) was done using the markings on the measuring pigtail. Dilatation of the channel was done with balloon catheters (Armada™, Abbott Laboratories) depending on pressure gradients; the required sizes were elected ranging from 6 × 60 to 10 × 60 mm. Subsequently, advancement of the sheath into the portal vein was performed. From here, deployment of the stent was conducted. If necessary, extension of the tract was done using bare stents (Absolute Pro™ Vascular, Abbott Laboratories) or the abovementioned balloon. Finally, the pressure is measured again using a riser tube. At the end of the procedure, a new central venous catheter was placed and in case of needed jugular puncture a full dose x-ray image was made to exclude accidental pneumothorax.

### Statistics

Where appropriate, a two-sided *t*-test was used. The level of significance was adjusted to *p* = 0.05; this was applied only for the hypothesis. Exploratory *t*-tests were done to compare the patients with hybrid interventions from those with conventional TIPS to identify bias. All calculations were done using R Project for Statistical Computing with the package “The great truth” [13].

## Results

### Study population

During the study period, we performed a total of 20 TIPS procedures outside regular duty hours as emergencies. Without exception, the indications were established on an interdisciplinary basis with the in-house hepatology department. Of these, ten interventions were performed as hybrid TIPS procedures and ten as conventional TIPS procedures. The average age of the patients was about 63 years. Eleven patients (55%) were male. Serum creatinine was elevated in nine patients (45%). Twelve patients (60%) had prolonged thromboplastin time. Thirteen patients (65) had an elevated INR (> 2). Seven patients (35%) were thrombocytopenic (< 50 G/L). Seventeen patients received a diagnosis of liver cirrhosis. The mean Meld score was 17.2 ± 8.75. Indications for TIPS procedure were acute variceal hemorrhage, acute hydropic decompensation, or acute Budd-Chiari syndrome. Some patients had more than one of these indications simultaneously. A detailed description and the respective proportions are given in Table 1. Three patients died within 7 days after the intervention. Regarding clinical parameters, patients who received a conventional TIPS procedure did not differ from patients who received a hybrid TIPS.

**Table 1:** Detailed description and the respective proportions

Description of the study population	
Mean age (years)	62.9 ± 18.7
Male	11 (55%)
Serum creatinine increased (1.35 mg/dL in men; 1.04 mg/dL in females)	9 (45%)
Increased partial thromboplastin time (> 35 s)	12 (60%)
Increased INR (> 2)	13 (65%)
Decreased platelet count (< 50.000 per µL)	7 (35%)
Diagnosed liver cirrhosis	17 (85%)
Meld Score	17.2 ± 8.75
Clinical indication for TIPS	
Acute variceal bleeding	8 (40%)
Acute hydropic decompensation (refractory to therapy)	10 (50%)
Acute Budd-Chiari syndrome	4 (20%)
Presence of hepatorenal insufficiency	4 (20%)
Presence of encephalopathy	4 (20%)
Procedure parameters and outcome	
Dose area product (cGy × cm^2^)	20,087 ± 32,460
Fluoroscopy time	35.1 ± 19.4
Duration of the whole intervention	88.2 ± 48.5
Pre-interventional portosystemic gradient (mmHg)	21 ± 4.41
Post-interventional portosystemic gradient (mmHg)	6.67 ± 4.53
Utilization of an additional proximal bare stent	3 (15%)
Death within 7 days after the intervention	3 (15%)

### Procedure parameters

Mean dose area product was 20,087 cGy × cm^2^. The average fluoroscopy time was 35.1 min. The average total intervention time was approximately 88.2 min. In the whole collective, the portosystemic gradient was 21 mmHg pre-interventional and 6.7 mmHg post-interventional. Thus, the gradient was significantly reduced in the overall collective (*p* < 0.001). A stent graft was used in all patients. In three patients, the stent had to be extended proximally. No intra-interventional complications occurred. All TIPS procedures were technically successful. No pneumothorax was observed.

### Effect of hybrid TIPSS—comparison of parameters of patients with and without hybrid TIPS

**Fig. 2:**
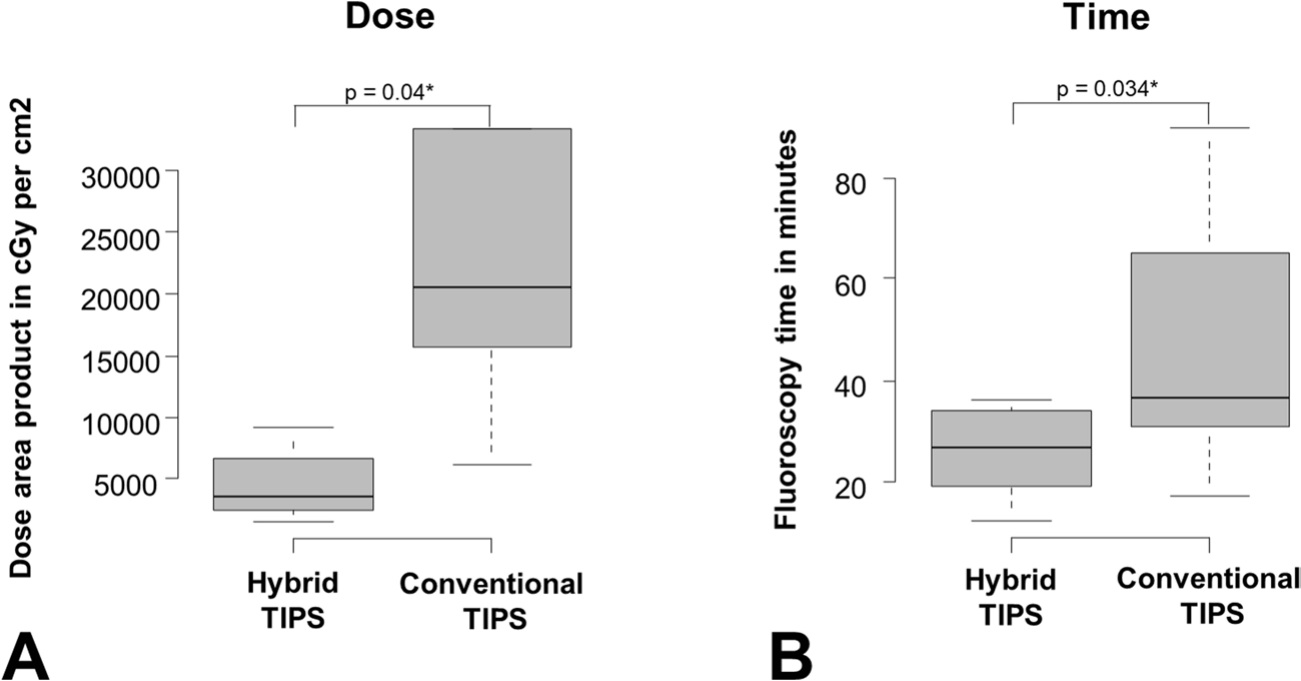
**A** The significantly reduced radiation exposure during hybrid TIPS and conventional TIPS assessed by the surrogate of the dose area product. **B** The significantly reduced fluoroscopy time of hybrid TIPS compared to conventional TIPS

Image fusion was considered helpful and correctly co-registered in all cases (10/10). Mean dose was significantly lower using angio-CT (4542 vs 35,633 cGy × cm^2^, *p* = 0.04). Fluoroscopy time was also significantly lower for this group (26 vs 45 min, *p* = 0.034). Further, the duration of the whole procedure was also significantly lower (65 min vs 112 min, *p* = 0.034). All patients who died within 7 days after the intervention were in the conventional therapy group, but this difference was not significant. A detailed description can be found in Table 2. Dose and fluoroscopy time are illustrated in Fig. 2.

**Table 2:** Detailed description of all patients who died within 7 days after the intervention in the conventional therapy group

	Conventional TIPS n = 10	Hybrid TIPS n = 10	*p*-value
Description of the study population			
Mean age (years)	68.5 ± 10.4	57.2 ± 23.7	0.19
Male	5 (50%)	6 (60%)	1
Serum creatinine increased (1.35 mg/dL in men; 1.04 mg/dL in females)	5 (50%)	4 (40%)	1
Increased partial thromboplastin time (> 35 s)	6 (60%)	6 (60%)	1
Increased INR (> 2)	6 (60%)	7 (70%)	1
Decreased platelet count (< 50.000 per µL)	4 (40%)	3 (30%)	1
Diagnosed liver cirrhosis	8 (80%)	9 (90%)	1
Meld Score	20.3 ± 10.9	14.1 ± 4.49	0.12
Clinical indication for TIPS			
Acute variceal bleeding	4 (40%)	4 (40%)	1
Acute hydropic decompensation (refractory to therapy)	3 (30%)	7 (70%)	0.18
Acute Budd-Chiari-Syndrome	3 (30%)	1 (10%)	0.58
Presence of hepatorenal insufficiency	3 (30%)	1 (10%)	0.58
Presence of encephalopathy	3 (30%)	1 (10%)	0.58
Procedure parameters and outcome			
Dose area product (cGy*cm^2^)	35,633 ± 40,994	4542 ± 2614	0.04*
Fluoroscopy time	44.5 ± 23.2	25.8 ± 8.12	0.034*
Duration of the whole intervention	112 ± 57.8	64.8 ± 20.3	0.034*
Pre-interventional portosystemic gradient (mmHg)	21.8 ± 5.09	19.9 ± 3.42	0.41
Post-interventional portosystemic gradient (mmHg)	8.55 ± 5.26	4.17 ± 1.2	0.053
Utilization of an additional proximal bare stent	2 (20%)	1 (10%)	1
Death within 7 days after the intervention	3 (30%)	0	0.21

^*^indicates significant results with p < 0.05

The mean dose length product of the CT examination was 121.7 ± 50.7 mGy × cm and the mean computed tomography dose index was 4.6 ± 1.5 mGy.

## Discussion

This study investigated the extent to which the TIPS procedure performed in hybrid mode with an angio-CT facilitates a single interventional radiologist under emergency conditions. The results show that (i) this method is technically feasible, (ii) it leads to a reduction of radiation exposure for the interventional team and (iii) subsequently results in shorter procedure times, and (iv) further, the results indicate higher patient safety compared with the procedure performed by fluoroscopy alone.

In general, image-guided puncture of the portal vein is recommended during TIPS procedure. The method of choice is sonographically guided puncture of the portal vein from within the hepatic vein. In this case, sonography is performed at baseline and then during the intervention. Sonographic guidance is then performed after the material has been placed in the hepatic vein and while the TIPS needle is advanced by an optimally experienced ultrasound examiner [3, 4]. Despite using this recommended technique, breathing movements of the patient and suboptimal ultrasound windows remain limiting factors. Furthermore, outside of regular duty hours, there may not be an additional experienced physician available to perform the sonography [5]. In this situation, it may be appropriate to use angio-CT to guide the puncture although it is not standard practice at the moment.

### Other methods than angio-CT

Modern angiography equipment allows 3D data from preliminary examinations to be imported from other devices and manufactures. This data can then also be displayed as fusion images on the procedure monitor, too. Although this type of fusion has not found its way into this study, problems arise from it in our experience. Perfect fusion is often difficult because, for example, ascites is more pronounced or less pronounced at the time of intervention than in the preliminary examinations. As a result, the position of the liver and therefore the portal vein is altered. Therefore, we believe that a CT scan and then immediate coregistration of the 3D data on the angiography table is beneficial [11, 12].

To acquire the 3D data of the portal vein for image fusion, a cone beam CT can also be performed pre-interventionally. Compared with the acquisition of data by dedicated CT, motion artifacts occur more frequently and, because of the significantly reduced contrast-to-noise ratio, helpful contrasting of the portal vein for subsequent segmentation via i.v. contrast administration is difficult [7, 8]. Further disadvantages of the dyna-CT are the small field of view as well as of center rotations [14].

In contrast, angio-CT registers the position of the portal vein in relation to the angiography table very accurately. For this reason, image fusion is dependent on patient cooperation and may therefore be disrupted if the patient is incompliant until finally the fusion cannot be used in a meaningful way. In this situation, the registration can be corrected manually but with limitations in accuracy and time. This was not necessary in the study population presented here; general intubation anesthesia for this method can be considered.

### Radiation dose

TIPS procedure using an angio-CT results in less radiation exposure from the angiography unit alone but is of course associated with performing a prior CT scan before the intervention starts. Even though the CT-dose values of this work were relatively low, a net reduction in radiation exposure for the patient is not necessarily to be expected for the overall procedure. However, there is certainly a reduction in radiation exposure for the intervention team, as they do not have to be present in the examination room at the time of the CT scan. Besides, the performed angio-CT of the portal vein as part of the hybrid intervention might render prior planning CTs redundant and therefore not increase radiation exposure of the patient at all.

### Practical aspects of the angio-CT

The use of angio-CT presents logistical challenges for the interventional team [9]. For example, explicit planning of the position of the anesthesia team and equipment, including cables and lines on the patient, is essential to ensure a smooth procedure. However, provided this is prepared and organized, a hybrid TIPS procedure appears to be superior to the conventional TIPS procedure using solely fluoroscopy guidance. Our results show a significant decrease of fluoroscopy time to a level which is similar to results of ultrasound-guided TIPS procedures [4]. Angio-CT is currently emerging development in interventional radiology. Nowadays, most manufacturers have a device of this type in their portfolio. The possibilities of its use are manifold and have yet to be developed and researched by us interventional radiologists. But technicians and interventionalists require training in both modalities, encompassing separate operational modes as well as hybrid mode, to effectively use the proposed method.

The procedure presented in this paper is only one of many ways to apply this device. In this application, it should be emphasized that it allows an intervention for a single interventional radiologist with safety, which normally recommended requires a second physician for the sonography [3, 4]. However, it should be mentioned that the operation of an angio-CT requires medical-technical radiology assistants. Performing TIPS with angio-CT might also be a cost driver because of the increased technical effort. To reduce the costs, a two-room installation of the angio-CT can be considered allowing the CT and the angio-suite to operate independently and only when required in hybrid mode. It should be considered that training of the personal is also required for post-processing and image fusion of the CT-VRT into the fluoroscopic image monitor. After training, post-processing and image fusion is no more than approx. 2 min, because CT images are automatically sent to a dedicated angio-CT console automatically generating VRTs. These must be tailored to the patient and to the contrast to a limited extent. A single button projects the reconstruction onto the fluoroscopy monitor and creates the image fusion. For this study, precise durations of the post-processing and image fusion were not measured.

In some cases, especially when ascites was present, we observed a slight offset between the CT scan or the 3D data, respectively, and the actual position of the portal vein. The reason for this is most likely a tilt of the sometimes very hard liver, due to the placement of the rigid foreign material in the hepatic veins. Ultimately, even with low offsets of the method proposed here, the main advantage is that the puncture attempts of the portal vein mainly take place in the correct area. A lateral projection of the beam path can also be applied for a.p. orientation, with the fused data rotating analogously on the live monitor; thus, anterior, or posterior correction of the needle tip can be targeted. In contrast, fluoroscopically guided puncture of the portal vein alone results in a series of punctures that may be well off-target but fully contribute to the patient’s procedural risk [3, 4].

The utilization of angio-CT in TIPS procedures can offer an effective option. Nonetheless, the decision to use this method should be carefully considered by an experienced interventional radiologist, taking into account the patient’s specific characteristics, available equipment, and institutional protocols. Optimal patient outcomes hinge upon close collaboration and communication among all clinical partners, including the radiologist, interventionalist, and other healthcare professionals. Ensuring thorough coordination throughout the entirety of the process allows for the most beneficial outcome for the patient.

### Limitations

This study is a single-center study with a relatively small study population. The patients have not methodically been randomized for the groups; the interventional radiologist dichotomized patients based on the estimated degree of difficulty of the procedure and the availability of the angio-CT. For difficult cases, the angio-CT was preferred; this further supports the here-presented results as those seemingly more difficult cases still exhibited lower radiation exposure and intervention times. The difference of the area dose product seems to be over-proportionally increased in the conventionally treated group comparing the difference of the fluoroscopy time. This might be caused by the subjective need for better image quality by the interventional radiologist in prolonged procedures with selecting more radiation-intensive setups. Furthermore, the groups have not been matched for their body mass index, nor was this parameter assessed. Lastly, the here-proposed method has not been compared to the standard method including recommended ultrasound guidance of the portal vein puncture by an experienced additional physician; this is also a limitation for the comparison of radiation doses of the procedural fluoroscopy. It should be mentioned that the relevantly reduced mortality (3/10 vs. 0/10) in the cohort of patients in whom hybrid TIPS was performed could be a statistical fluctuation since the level of significance was not reached.

## Conclusion

TIPS procedure using Angio-CT is superior to mere fluoroscopy guidance. The hybrid intervention is faster and has lower radiation exposure for the intervention team. In addition, the results of this work suggest increased patient safety. If only one interventional radiologist is available for the TIPS procedure, this intervention should be performed using angio-CT whenever available.

## Abbreviations

cGy: Centigray
cm: Centimeters
CT: Computed tomography
F: French
G: Gauge
G/L: Giga per liter
i.v.: Intravenous
INR: International normalized ratio
IVC: Inferior vena cava
kV: Kiloelectronvolts
L: Liters
mGy: Milligray
ml: Milliliters
mm: Millimeters
mmHg: Millimeters of mercury
MRI: Magnetic resonance imaging
s: Seconds
TIPS: Transjugular intrahepatic portosystemic shunt
VRT: Virtual rendering technique

## Declarations

### Guarantor

The scientific guarantor of this publication is PD Dr. Jonathan Nadjiri, EBIR.

### Statistics and biometry

One of the authors has significant statistical expertise. Further, no complex statistical methods were necessary for this paper.

### Methodology

• retrospective

• case–control study

• performed at one institution
